# Mutations in Genes Producing Nitric Oxide and Hydrogen Sulfide and Their Connection With Apoptotic Genes in Chronic Myeloid Leukemia

**DOI:** 10.7759/cureus.61570

**Published:** 2024-06-03

**Authors:** Bahaaddin A Saber, Ashabil Aygan, Abbas Salihi

**Affiliations:** 1 Department of Bioengineering and Sciences, Faculty of Applied Sciences, Kahramanmaraş Sütçü Imam University, Kahramanmaraş, TUR; 2 Department of Biology, Faculty of Applied Sciences, Kahramanmaraş Sütçü Imam University, Kahramanmaraş, TUR; 3 Department of Biology, College of Science, Salahaddin University-Erbil, Erbil, IRQ

**Keywords:** next-generation sequencing (ngs), sanger sequencing, next generation sequencing (ngs), chronic myeloid leukaemia, nos3 gene, cth gene

## Abstract

Background

Despite advances in chronic myeloid leukemia (CML) genetics, the role of nitric oxide (NO) and hydrogen sulfide (H_2_S) gene mutations and their relationship to apoptotic genes is unclear. Therefore, this study investigated NO- and H_2_S-producing genes' mutations and their interactions with apoptotic genes using Sanger sequencing and next-generation sequencing (NGS).

Methodology

A complete blood count (CBC) was carried out to measure the total number of white blood cells, while IL-6 levels were assessed in both control and CML patients using an ELISA technique. Sanger sequencing was used to analyze mutations in the *CTH* and *NOS3* genes, whereas NGS was applied to examine mutations on all chromosomes.

Results

White blood cell (WBC) and granulocyte counts were significantly higher in CML patients compared to controls (*p*<0.0001), and monocyte counts were similarly higher (*p*<0.05). Interleukin-6 (IL-6) levels were significantly elevated in CML patients than controls (*p*<0.0001), indicating a possible link to CML etiology or progression. Multiple mutations have been identified in both genes, notably in *CTH* exon 12 and the *NOS3* genes VNTR, T786C, and G894T. This study also measured IL-6 concentrations using IL-6 assays, identifying its potential as a CML prognostic diagnostic. WBC counts, granulocyte counts, and mid-range absolute counts, or MID counts, were significantly higher in CML patients than in normal control individuals. NGS identified 1643 somatic and sex chromosomal abnormalities and 439 actively expressed genes in CML patients. The findings imply a genomic landscape beyond the *BCR*-*ABL1* mutation in CML development compared to other databases.

Conclusion

In conclusion, this study advances the understanding of the genetic characteristics of CML by identifying mutations in the NO- and H_2_S-producing genes and their complex connections with genes involved in apoptosis. The comprehensive genetic profile obtained by Sanger sequencing and NGS provides possibilities for identifying novel targets for therapy and personalized treatments for CML, therefore contributing to developments in hematological diseases.

## Introduction

Chronic myeloid leukemia (CML), also known as chronic myelogenous leukemia, is a myeloproliferative neoplasm that involves uncontrolled myeloid cell growth [1]. CML differs from other myeloproliferative neoplasms because of the *BCR-ABL1* fusion gene and Philadelphia chromosome (Ph) caused by t(9;22)(q34.1;q11.2) [2-4]. Proven (1805) genes from every leukemia subtype have been used to develop the database of leukemia gene literature, or dbLGL [5].

Hydrogen sulfide (H_2_S) is synthesized internally inside mammalian tissues by the enzymatic actions of cystathionine-β-synthase (CBS), cystathionine γ-lyase, and 3-mercaptopyruvate sulfurtransferase, which is located in the mitochondria. The mechanism regulates the vascular diameter, and protects the endothelium against oxidative stress, ischemia, reperfusion damage, and chronic inflammation, by activating potassium (K^+^) channels in vascular smooth muscle cells [6,7]. In mammals, nitric oxide synthase (NOS) in tissues generates NO. Neuronal, inducible, and endothelial NOS enzymes convert L-arginine to NO. Endothelial and neuronal NOS (eNOS and nNOS) are constitutive and calcium-dependent isoforms with small NO production. Conversely, the calcium-independent inducible NO synthase (iNOS) may be continually activated. The NO response depends on NO concentrations; lower NO concentrations stimulate cellular growth and suppress apoptosis, whereas greater NO concentrations stop the cell cycle and induce apoptosis [7-12].

Despite developments in CML genetics, little is known about NO and H_2_S gene mutations and their connections with apoptotic genes. To understand CML genetics, NO and H_2_S gene mutations and their interactions with apoptotic genes must be studied. This study will explain CML's molecular processes and the complex connection between these mutations and apoptotic genes. This study used Sanger sequencing and next-generation sequencing (NGS) to create a detailed genetic profile of CML, which could lead to novel therapeutic targets and personalized therapies for this hematological disorder.

## Materials and methods

Sample collection

Blood samples were collected from 40 CML patients and 40 healthy individuals and placed into ethylene diamine tetraacetic acid (EDTA) tubes. Each tube received 3 ml of sodium citrate for haematology tests, and 3 ml was placed in gel tubes to induce coagulation and collect blood for interleukin (IL) measurement.

Complete blood count

The blood sample was analysed using a Coulter counter (Medonic M16M and M16 models; CLIAwaived Inc., CA, USA) to determine the total white blood cell (WBC), lymphocyte, and monocyte counts.

DNA extraction and quantification

The extraction of genomic DNA from blood samples collected from persons diagnosed with CML was performed using the genomic blood DNA isolation kit (Hibrigen, Turkey) according to the manufacturer's instructions, with some modifications. In summary, blood samples were obtained and promptly handled within a specified time period to avoid DNA deterioration. After extracting the DNA, we assessed both the amount and the quality of the isolated genomic DNA. The DNA concentration was measured using a nanodrop spectrophotometer at a wavelength of 260 nm. In addition, the quality of the extracted DNA was assessed by determining the A260/A280 ratio. A ratio between 1.8 and 2.0 indicates that the DNA is free of contaminants and lacks any protein or other impurities. Only DNA samples with A260/A280 ratios within the acceptable range were selected for downstream applications, ensuring high-quality genomic DNA for further molecular analysis.

Determination of genotype

Three genetic variants within the *NOS3* gene and one variant of the *CTH* gene were studied. Individual amplification of DNA for each variant was performed using polymerase chain reaction (PCR), followed by gel electrophoresis and sequencing analysis. DNA sequencing plays a vital role in understanding genetic diversity and uncovering potential health and disease susceptibility implications. The PCR product underwent sequencing, particularly Sanger sequencing. Initially, the sample sequence was processed at the Kahramanmaraş Sütçü Imam University, ÜSKIM Laboratory, following purification and amplification with specific primers for both directions. Subsequently, a sequencing library was created using the Applied Biosystems ABI 3100 AVANT DNA Sequencer (Thermo Fisher Scientific Inc., Waltham, MA) to enable thorough sequencing analysis. The resulting extension file (AB1) was then scrutinised using Mutation Surveyor software, version 5.2.0 (SoftGenetics, State College, PA) to detect any mutations or variations in the target sequence.

NGS has transformed genomics by granting scientists unparalleled access to extensive genetic information. An essential stage in this procedure involves preparing the sequencing library, which entails converting the desired DNA into a suitable format for the sequencing platform. For this reason, we transferred the DNA samples to the Istanbul Laboratory, in Istanbul, Turkey. After checking the quality and purity of the DNA samples through nanodrop analysis, we proceeded to the next library preparation step. The library preparation process typically commences with fragmentation of the target DNA, followed by adapter ligation and PCR amplification. During the library preparation and sequencing process, numerous sequence artefacts negatively affect raw data quality for downstream analyses. Therefore, quality control and preprocessing of the raw data are crucial steps to ensure the accuracy and reliability of the sequencing results. Various tactics, such as paired-end and mate-pair sequencing, can be applied, which help the assembly of short sequences into contigs and scaffolds. After preparing the library through the standard protocols, we conducted the sequencing step using the DNBSEQ-G400 flexible genome sequencer (MGI Tech Co., Ltd, Thailand), created based on a new flow cell system that could flexibly assist a range of various sequencing modes. The raw data was analyzed using the SAMtools software (Sanger Institute, Cambridgeshire, UK), and then compared with external databases (such as gnomAD, COSMIC, and cBioPortal) to annotate and visualize the results. The subsequent data analysis involved several steps: quality control, read mapping, variant calling, and annotation.

IL-6 measurement

IL-6 levels in the study samples were measured with a particular kit (catalogue no. DE4640; Demeditec Diagnostics, Kiel, Germany). The concentration was calculated using the Stat Fax ELISA reader (Awareness Technology, Inc., Palm City, FL), and statistical analysis was performed using GraphPad Prism, version 10 (GraphPad Software, Inc./Dotmatics, Boston, MA) after establishing a standard curve using MyAssays software (MyAssays Ltd., Brighton, UK). All measurements were taken in triplicate following the manufacturer's recommendations to ensure accuracy. Furthermore, thorough quality control methods were implemented throughout the experimentation phase to validate the results acquired.

Statistical analysis

Comparisons between patients with CML and healthy individuals were performed using an unpaired t-test, and values were presented as means±SEMs. The graphics, computations, and statistical analyses were generated using GraphPad Prism, version 10. A *p*-value of <0.05 was considered statistically significant.

Ethical considerations

Ethical considerations concerning the collection of human blood samples for research purposes were addressed in accordance with the Declaration of Helsinki. The study was approved by the Human Ethics Research Committee of the College of Science, Salahaddin University-Erbil (under reference number 4/5/439). All patients provided an informed consent to allow their blood samples to be examined.

## Results

Complete blood count

**Figure 1:**
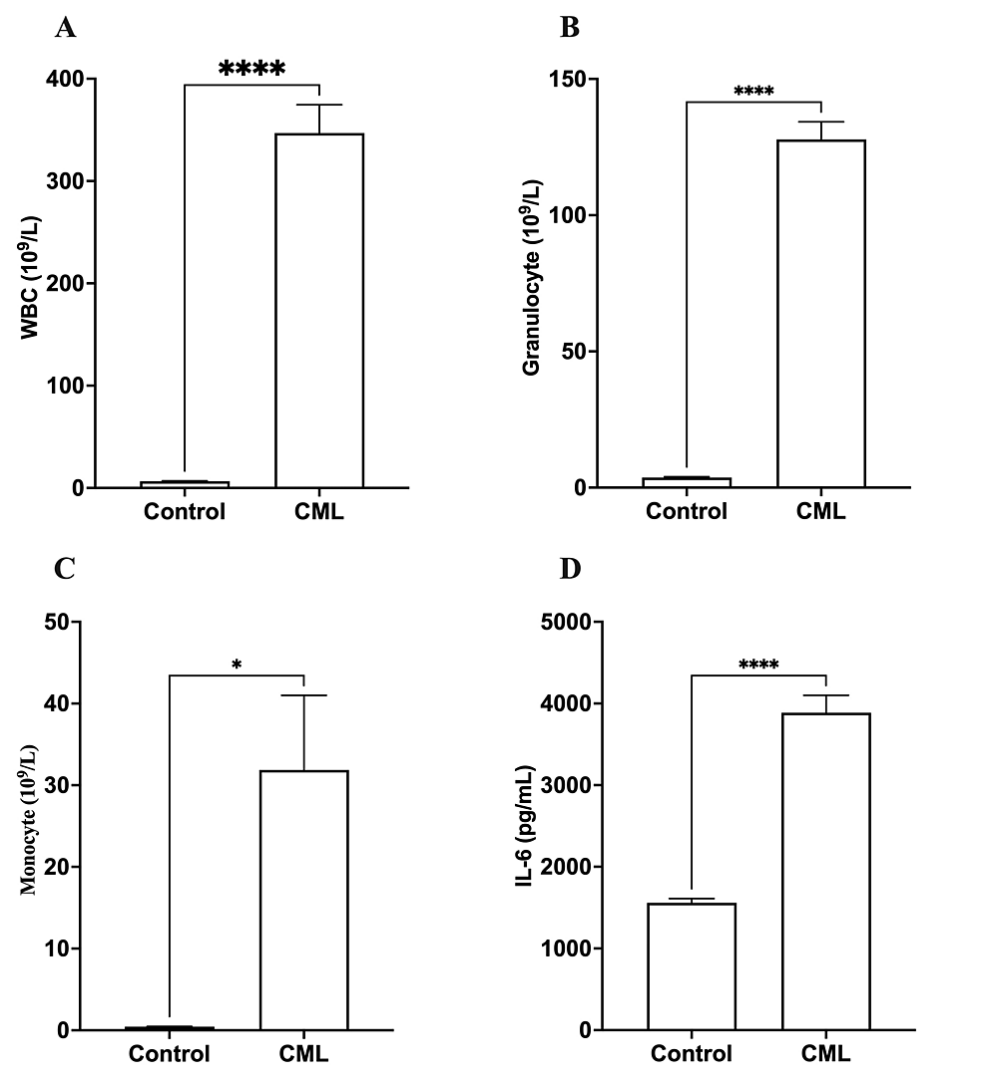
A comparison of hematological parameters and IL-6 levels between controls and chronic myeloid leukemia (CML) patients Patients with CML had significantly greater (*p*<0.001) total WBC (A) and granulocyte counts (B). (C) Patients with CML had a significantly higher monocyte count (*p*<0.05). (D) In CML patients, IL-6 levels were significantly higher (*p*<0.001). *p<0.05; ****p<0.0001 vs. healthy individuals

The results indicated significant differences between the control group and CML patients in all examined parameters, including WBC count, granulocyte count, monocyte count, and IL-6 levels. Patients with CML showed significantly higher (*p*<0.0001) WBC and granulocyte counts than the control group. In addition, monocyte counts were considerably greater (*p*<0.05) in individuals with CML. Still, the difference was not as noticeable as in WBC and granulocyte counts, as shown in Table 1 and Figures 1A-1C.

**Table 1:** A comparison of hematological parameters and IL-6 levels between controls and chronic myeloid leukemia (CML) patients Patients with CML had significantly greater (*p*<0.001) total WBC and granulocyte counts. Patients with CML had a significantly higher monocyte count (*p*<0.05). In CML patients, IL-6 levels were significantly higher (*p*<0.001).

Parameters	Control	CML	*p*-value
WBC (10^9^/L)	6.56±0.38	346.9±27.66	0.0001
Granulocyte (10^9^/L)	3.68±0.288	127.8±6.448	0.0001
Monocyte (10^9^/L)	0.455±0.035	31.87±9.141	0.05
IL-6 (pg/mL)	1558±53.5	3888±212.8	0.0001

IL-6 concentration

Patients with CML had markedly increased (*p*<0.0001) levels of IL-6 compared to control individuals, indicating that IL-6 may have a role in the onset or progression of CML, as shown in Table 1 and Figure 1D.

Sanger sequencing

**Figure 2:**
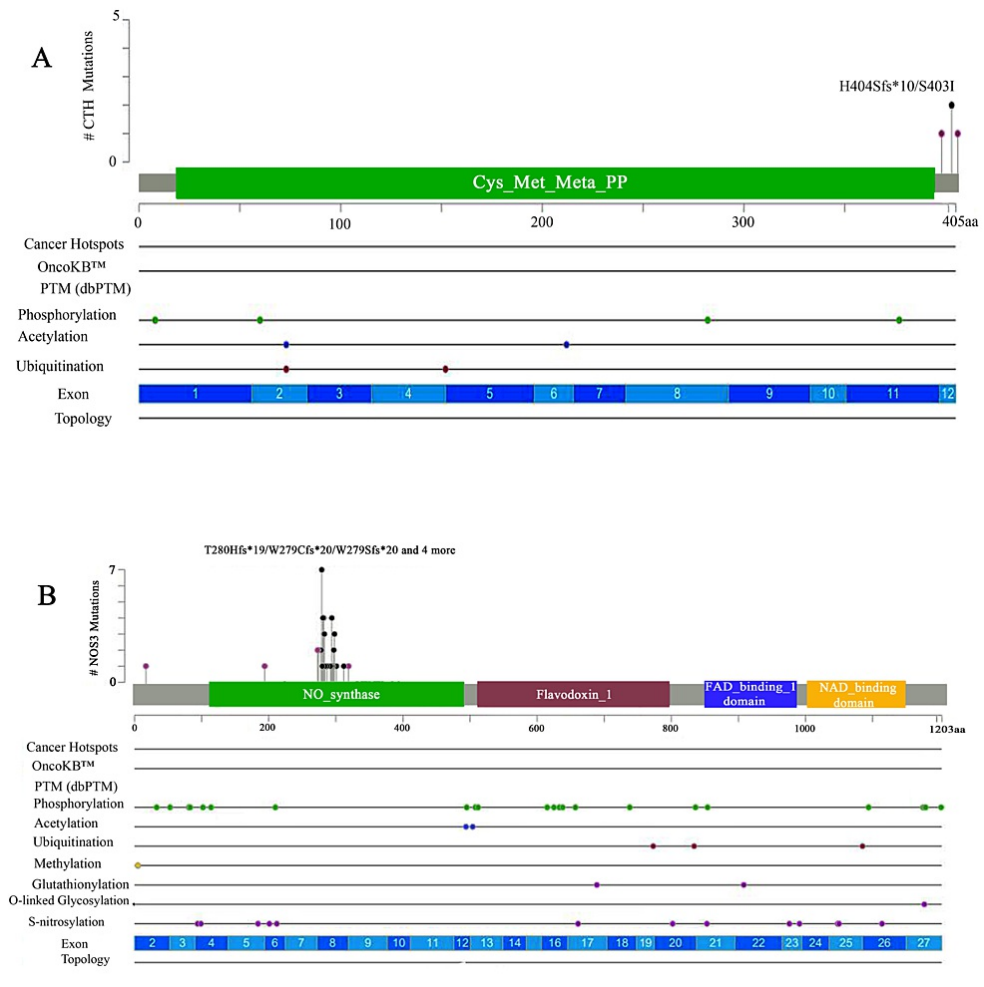
Sanger sequence analysis through the cBioPortal database (A) A lollipop mutational map showing the CTH gene mutation. (B) A lollipop mutational map showing the NOS3 gene (VNTR, T786C, and G894T). PTM, post-translational modification

**Figure 3:**
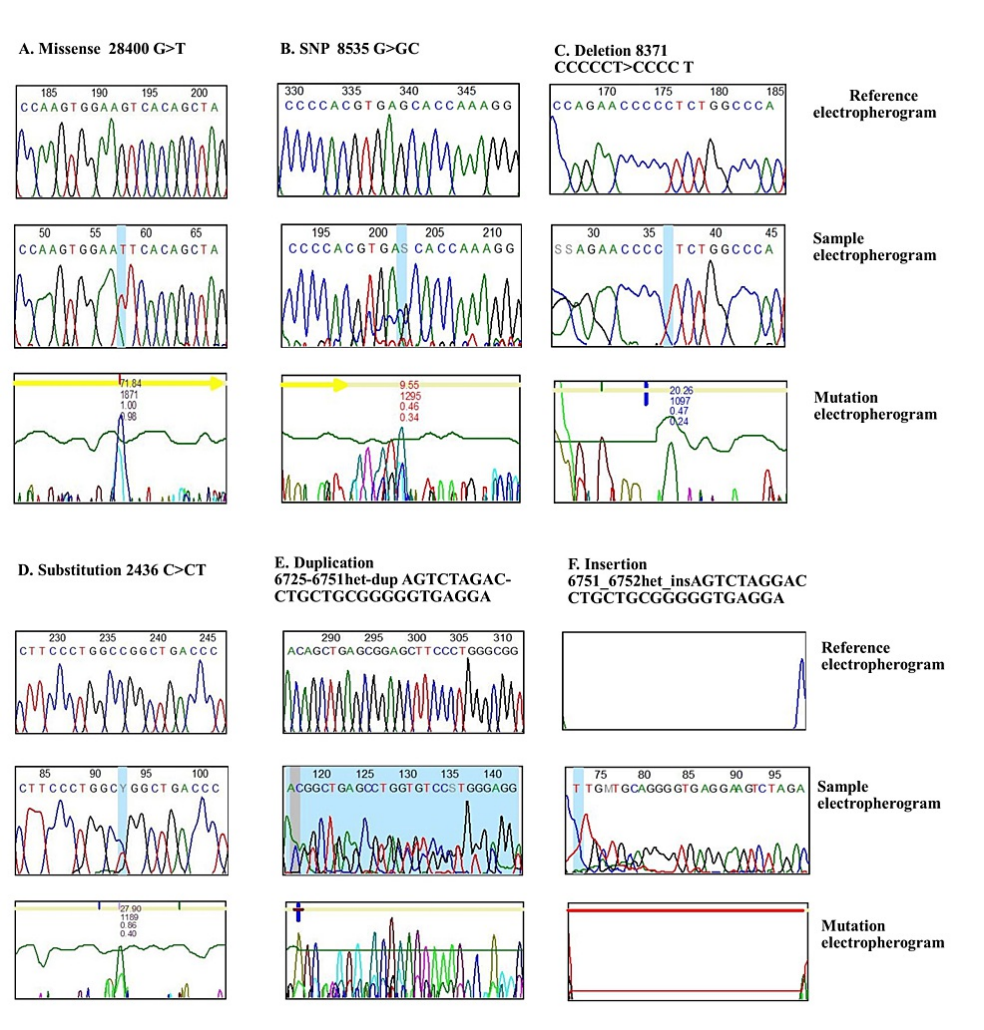
Electropherograms showing the mutational sample with reference (A) A Sanger sequence chromatogram for the CTH gene showing the missense mutation (dbSNP:1021737), and amino acid change (serine>isoleucine) in position (28400G>GT); (B) a Sanger sequence chromatogram for the NOS3 gene showing the mutation in the splice region on T786C that changes the nucleotide (8535G>GC); (C) a Sanger sequence chromatogram for the NOS3 gene showing the mutation in the splice region on T786C that changes the nucleotide (8371 C>T); (D) a Sanger sequence chromatogram for the NOS3 gene showing the substitution mutation (dbSNP:2070744) that changes the nucleotide (2436C>T) located on G894T; (E) a Sanger sequence chromatogram for the NOS3 gene showing the duplication mutation 6725_6751het_dupAGTCTAGACCTGCTGCG GGGGTGAGGA) located in VNTR; (F) a Sanger sequence chromatogram for the NOS3 gene showing the duplication mutation 6751_6752het_INSAGTCTAGGACCTGCTGCGGGGGTGAGGA) located in VNTR.

CML mutations were found in 40 *NOS3* and *CTH* gene-sequenced CML patients compared to external databases (gnomAD, COSMIC, and cBioPortal). *CTH* determined exon 12 missense, substitution, inversion, and duplication mutations (Figure 2a). All missense genes (1:70904800) replicated in multiple patients, and heterozygous mutations (28400G>GT) led to amino acid changes (serine>isoleucine) (dbSNP:1021737), and all mutations were at the end of the cys_met_meta_pp domain, as shown in Figure 3A and Appendix A.

Additionally, the *NOS3* gene, which was sequenced using three primers (VNTR 4a/b, T786C, and G894T), found numerous mutations in different locations on the gene (Figure 2b) when compared to external databases (gnomAD, COSMIC, and cBioPortal). The T786C and G894T mutations were located in the NOS3 gene domain, whereas the VNTR change occurred in intron 3 of all T786C patients. These mutations included missense, substitution, synonymous, splice region, and intron mutations. In addition, the (dbSNP:1799983) variant present in many samples had a missense mutation that changed the nucleotides (8468T>TG) on the position of (7:150696111), which replicated in many samples. The other three mutations in the splice region were (7:150696187, 7:150696176, and 7:150696178) and the variants (8533, 8535G>GC, and 8544G>GA) (Figure 3b; Appendix B). However, the G894T primers were sequenced, and different types of variations were estimated, including modifications to nucleotides that mutated sequences, and substitution mutations in all patients. The 21 (dbSNP:2070744) was found through the nucleotide variants (2436C>T) on (7:150690079) (Figure 3d; Appendix C). The VNTR modification was also on *NOS3*, and all variations that altered nucleotides included mutation types such as substitution, duplication, and insertion. The variant (dbSNP:3918168) is produced by a nucleotide change (6714G>GA) at location (7:150694357). This variant also resulted in duplication and insertion mutations, including (6725_6751het_dupAGTCTAGACCTGCTGCG GGGGTGAGGA) at locations (7:150694368_7:150694394). The VNTR also had an insertion mutation due to a changed nucleotide (6751_6752) (Figure 3e; Appendix D).

Next-generation sequencing

**Figure 4:**
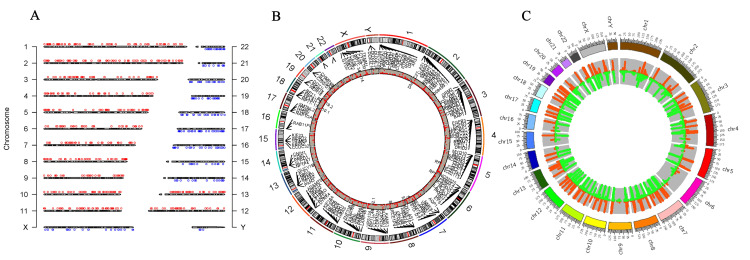
Next-generation sequencing (NGS) analysed through the SRplot database (A) The chromosome distribution map illustrates the highest concentration of chromosomes; (B) a two-dimensional Circos plot displaying four columns, with the first indicating chromosomes, the second showing starting coordinates, the third indicating end coordinates, and the fourth representing fold change reflecting gene upregulation and downregulation during cancer progression and GC content variability; (C) an RCircos diagram (version 1.2.2, an R package for Circos 2D track plots) depicting gene names to showcase expressed genes and copy number variation, and includes information on chromosome location in the first three columns, along with gene name locations, while log2fc is displayed in another column.

Next-generation whole-genome sequencing identified 1643 somatic and sex chromosomal abnormalities and 439 gene expressions in CML patients. The results were cross-referenced to the gnomAD, COSMIC, and cBioPortal databases. Patients with CML expressed 439 genes. Figure 4A shows how all chromosomes contribute to CML. Specifically, the X chromosome carries 96 of the 106 sex differences. Ninety-four intron alterations occur during gene expression, including upregulation and downregulation. Figure 4C shows the genes *CXorf36*, *ASB11*, *ZRSR2*, and *TENM1*. The remaining two mutations (out of 96) are unidentified. There are 10 chromosomal Y variants in four genes' intronic regions. Furthermore, chromosome 1 has 98 mutations. There are 69 mutations in 29 expressed genes, and 29 remain unidentified. Among the 69 mutations, *CHD1L*'s frameshift-deletion mutation and *PIK3CD*'s splice region variation stand out. Finally, 67 of the 69 variations are introns. Furthermore, chromosome 2 had 163 alterations, with 95 in 44 expressed genes. The non-transcription region (AC012363.8) had 14 mutations at the same location as *MTND4P26*, whereas *EMILIN1* gained a missense mutation. There were also mutations in the GCA gene's intron and 3' untranslated region (3'UTR). There were 53 more unidentified mutations, including 15 in non-coding areas (Table 2).

**Table 2:** Variation distribution on genes and chromosomes

Chromosome no.	Mutation no.	Gene no.
X	96	12
Y	10	4
1	98	30
2	158	44
3	121	28
4	81	14
5	87	27
6	43	14
7	141	25
8	101	17
9	68	17
10	72	27
11	83	30
12	91	23
13	41	13
14	25	13
15	36	8
16	35	15
17	68	25
18	44	12
19	53	19
20	29	6
21	23	8
22	44	11

Furthermore, chromosome 3 revealed 121 mutations in 28 expressed genes. These 34 mutations remain unidentified, while the other two were synonymous and located on the *NPRL2* gene's *PLCL2* non-coding transcript exon. The *FLNB* and *TBCCD1* genes had two identical missense mutations. The remaining alterations were intron-based. Another 14 genes with 81 variations were found on chromosome 4. There were approximately 32 unidentified mutations, totaling 49. Two synonymous mutations and one missense *FGFR3* mutation were identified. The remaining alterations affected genes located in introns. Of the 87 mutations on chromosome 5, 40 were unidentified mutations. Figure 4b shows 47 of the 87 variations on 27 expressed genes. This variant had a 3'UTR mutation and a *CDH9* gene missense mutation. The *RP11-232L2.1* gene had exons that did not code for proteins. PPP2R2B's 5' untranslated region (5'UTR) and *GM2A*'s frameshift mutation were also identified; intronic mutations occurred. There were 14 active genes on chromosome 6. These genes contained 43 variations, including 24 unidentified mutations. The remaining 19 mutations were distributed among 14 genes, with six occurring in similar numbers on RP11-288G3.4 and *HLA-V*'s non-coding transcript exons. Mutations to the *HLA*-*DOA* splice region and the *TULP4* 3'UTR were also identified. The remaining genes were introns.

Chromosome 7 had a total of 141 different variations. Out of 141 occurrences, 79 were characterized by unidentified mutations, while 62 were associated with 25 specific genes. This investigation identified three mutations in the 3'UTR of the *AQP1* gene. We also detected two *MUC12* and *SMO* missense mutations, two *STRIP2* 3'UTR mutations, and a *CEP41* mutation. All the remaining ones were introns.

Furthermore, chromosome 8 contained a total of 101 genetic variations; 71 variations were not known and 30 variants had an impact on 17 genes being expressed. Both genes included equal quantities of non-coding transcript exons *SMARCE1P4* and *RP11-468O2.1*. The 5'UTR of the *CTSB* gene had one mutation, whereas the other mutations were located in the introns. Of the 68 variants found on chromosome 9, 17 were linked to actively expressed genes. There were at least 24 variants that contained mutations whose identity was not known, whereas 44 variations had mutations that had been identified. The 44 variations consisted of two missense mutations in the *KANK1* gene, three mutations in the 3'UTR of the *CDKN2A* gene, a mutation in a non-coding transcript exon of the *CCL27* gene, a missense and synonymous mutation in the SURF6 gene, and three mutations in the 3'UTR of the *MED22* gene. The most severe mutations were found in introns. The dataset contained a total of 72 mutations located on chromosome 10. There were 22 variations with unidentified alterations, whereas 50 variants were associated with 27 genes. Three mutations were detected in the 3'UTR of the *VPS26A* gene, whereas the other modifications were inside introns. Out of 83 variations, 26 were linked to mutations on chromosome 11 that were now unidentified. Of the 57 modifications, 30 were associated with expressed genes in intronic regions. However, there were three missense mutations in the *PIDD1* gene and three missense and synonymous mutations in the *MUC6* gene. Chromosome 12 contained a total of 91 genetic variants. Approximately 36 changes were associated with unidentified mutations, while the remaining mutations were associated with 24 functional genes. The *KANSL2* gene harbored two synonymous alterations in its 3'UTR. *ITGA7* and *NEMP1* had missense mutations, whereas *HOXC*-*AS3* had a mutation in a non-coding transcript exon.

Additionally, chromosome 13 had 41 variants. Twenty variants were related to unexplained mutations, whereas 13 to intron mutations in four expressed genes: *TPTE2*, *PAN3*, *RXFP2*, and *LMO7*. Chromosome 14 had 25 variants, of which two were unknown mutations. Except for synonymous *NEK9* mutations, the remaining 23 variants corresponded to 13 intron-expressed genes. Chromosome 15 had 36 variants, four unexplained mutations, and nine related to expressed genes. All of these changes were intronic except for an *MTHFS* gene missense mutation. Unknown mutations accounted for 16 of the 35 variants on chromosome 16. The other 19 variants affected nine intron-region genes, including *JPT2*, *TRAF7*, and *PLCG2*.

Chromosome 17 had 68 variations. Among these variations, 13 were linked to unknown mutations, while 55 were related to 25 expressed genes. The *ITGAE* gene had a frameshift mutation, and the *SMTNL2* and *CHRNE* 3'UTRs were also mutated. *ULK2* had a synonymous mutation, whereas *WIPF2* had both insertion and synonymous mutations. Intronic areas were mutated again. Chromosome 18 had 44 variations. The introns of 12 expressed genes (*AKAIN1*, *CDH2*, and *CDH7*) exhibited 33 variants. The remaining variations were unknown. Also, 26 of the 53 chromosome 19 mutations contained unknown mutations. An additional 27 variants were related to 19 expressed genes. The *WDR18* gene had a synonymous mutation, but *DOT1L* and *TDRD12* had two missense variations. Intron variation continued. Of the 29 chromosome 20 variants, 18 were associated with unknown mutations. This includes 11 changes to the intron regions of six expressed genes. The *MIR646HG* gene contained just one non-coding transcript exon mutation. Chromosome 21 had 23 variants, two related to unexplained mutations. Except for two *C2CD2* and* PDXK* 3'UTR mutations, the other 21 variations were found in eight expressed genes. All of these variants were found in introns. Chromosome 22 had 44 mutations, 13 of which were unknown. Another 31 variations were linked to 11 *BCR*-expressed genes and a mutation in *FOXRED2*'s intronic 3'UTR, as shown in Appendix E.

## Discussion

Elevated WBC counts are commonly observed in individuals diagnosed with CML [13]. The cost-effective and direct approach for detecting CML involves utilizing differential analysis and CBC techniques [14]; the parameter of CBC generally changes during cancer incidence [15] and also after chemotherapy administration [16]. During the occurrence of cancer, an increase in the total WBC count is observed. It is possible that, following treatment, the WBC counts subsequently decrease. Due to this rationale, the WBC count obtained via the CBC test has emerged as a biomarker for the detection of leukemia. This study observed a high total WBC count, granulocyte count, and MID count.

IL-6 has been postulated as a potential prognostic marker for CML [17]. As a result, IL-6 levels may rise significantly throughout CML, exceeding the baseline rate. The acquired findings were statistically significant, demonstrating an increase in IL-6 levels with the onset of cancer.

The nucleotide sequences of the *NOS3* and *CTH* genes were determined using Sanger sequencing. In CML patient samples, different changes were found in the *CTH* gene. These changes were all found outside the cys-met-meta-pp domain on exon 12. However, to our knowledge, no previous study has found a relationship between the *CTH* gene and CML, and this is the first study to show an extensive number of mutations in the *CTH* gene [18].

Furthermore, the *NOS3* gene exhibited distinct mutations in the *VNTR*, *T786C*, and *G894T* genes in colorectal cancer [19]. Notably, all these variants were found within the *NOS3* gene, except for specific variants in the *VNTR*. Together with the tyrosine kinase activator and *BCR*-*ABL1* genes [20], these results show that the *NOS3* gene is expressed in people with leukemia.

Many genetic disorders and syndromes have been identified in recent decades using NGS technologies. The utilization of NGS is rapidly becoming standardized as a diagnostic tool and for molecular patient monitoring, enabling the evaluation of treatment effectiveness [21]. The present study's findings indicate that 1643 variations were seen across the 22 chromosomes, including the XY chromosome. Furthermore, gene expression analysis revealed that 439 genes were actively expressed. Additionally, two genes were sequenced using the Sanger method, while one gene was identified using the ELISA technique. Nevertheless, the findings indicate that, apart from *BCR*-*ABL1*, several genes are linked to CML development.

A few study limitations may impact the ability to adapt to and understand the results. The study's sample size may not represent the CML population, limiting its external validity. The study's approach relies primarily on observational and genetic analysis, which may introduce biases or confounding factors that are not adequately controlled and addressed.

## Conclusions

The study thoroughly investigated the genetic landscape of CML, revealing insights into the delicate interaction between NO, H_2_S, gene mutations, and apoptotic genes. The *NOS3* and *CTH* gene mutations were identified using Sanger sequencing and NGS, indicating novel interactions with CML pathogenesis. The study found previously unknown mutations in the *CTH* gene and expanded the understanding of its role in CML. Additionally, various mutations in the *NOS3* gene, such as the *VNTR*, *T786C*, and *G894T* variations, revealed CML's complex genetic landscape. The NGS study found 1643 somatic and sex chromosomal abnormalities and 439 actively expressed genes, revealing CML's genomic complexity beyond the well-known *BCR*-*ABL1* mutation. These findings highlight the potential of NGS as a diagnostic and prognostic tool, providing insights into personalized treatment approaches for CML that extend beyond *BCR*-*ABL1* targeting strategies.

## Appendices

Appendix A

**Table 3:** CTH gene variation with amino acid change in chronic myeloid leukemia (CML)

Gene	Chromosome position	Mutation	Mutation genotype	Heterozygous/homozygous	Variants	Variant percentage	Amino acid change	External database
CTH								
	1:70904757	Substitution	T>TA	Heterozygous	28357T>TA	5.0%	None	Not Found
	1:70904800	Missense	G>GT	Heterozygous	28400G>GT	35.0%	Serine>Isoleucine	dbSNP:1021737
	1:70904800	Missense	G>GT	Heterozygous	28400G>GT	35.0%	Serine>Isoleucine	dbSNP:102173
	1:70905047	Inversion	G>GA	Heterozygous	28647G>GA	5.3%	None	Not Found
	1:70904800	Missense	G>GT	Heterozygous	28400G>GT	35.0%	Serine>Isoleucine	dbSNP:1021737
	1:70904800	Missense	G>GT	Heterozygous	28400G>GT	35.0%	Serine>Isoleucine	dbSNP:1021737
	1:70905019	Duplication	A	Heterozygous	28619het_dupA	5.0%	None	Not Found
	1:70904810	Substitution	A>AG	Heterozygous	28410A>AG	5.0%	Glycin>Glycin	Not Found
	1:70904811	Substitution	T>TC	Heterozygous	28411T>TC	5.3%	None	Not Found
	1:70905018	Substitution	T>TA	Heterozygous	28618T>TA	5.0%	None	Not Found
	1:70904770	Substitution	C>CT	Heterozygous	28370C>CT	5.3%	None	Not Found
	1:70904772	Substitution	C>CG	Heterozygous	28372C>CG	5.0%	None	Not Found
	1:70904800	Missense	G>GT	Heterozygous	28400G>GT	35.0%	Serine>Isoleucine	dbSNP:1021737
	1:70904758	Substitution	A>AT	Heterozygous	28358A>AT	4.8%	None	Not Found
	1:70904800	Missense	G>T	Homozygous	28400G>T	35.0%	Serine>Isoleucine	dbSNP:1021737
	1:70905045	Inversion	T>TA	Heterozygous	28645T>TA	5.0%	None	Not Found
	1:70905047	Inversion	G>GA	Heterozygous	28647G>GA	5.3%	None	Not Found
	1:70904810	Substitution	G>GC	Heterozygous	28410G>GC	4.3%	Valine>Tyrosine	Not Found
	1:70904800	Missense	G>GT	Heterozygous	28400G>GT	35.0%	Serine>Isoleucine	Not Found
	1:70905047	Inversion	G>GA	Heterozygous	28647G>GA	5.3%	None	Not Found
	1:70904758	Substitution	A>AT	Heterozygous	28358A>AT	4.8%	None	Not Found
	1:70905046	Inversion	T>TA	Heterozygous	28646T>TA	5.0%	None	Not Found
	1:70905047	Inversion	G>GA	Heterozygous	28647G>GA	5.3%	None	Not Found
	1:70904810	Substitution	G>GC	Heterozygous	28410G>GC	4.3%	Valine>Tyrosine	Not Found
	1:70904770	Substitution	C>CT	Heterozygous	28370C>CT	5.3%	None	Not Found
	1:70905019	Duplication	A	Heterozygous	28619het_dupA	5.0%	None	Not Found
	1:70905018	Substitution	T>TA	Heterozygous	28618T>TA	5.0%	None	Not Found
	1:70905046	Inversion	T>TA	Heterozygous	28646T>TA	5.0%	None	Not Found
	1:70904811	Substitution	T>TC	Heterozygous	28411T>TC	5.3%	None	Not Found
	1:70904758	Substitution	A>AT	Heterozygous	28358A>AT	4.8%	None	Not Found
	1:70905018	Substitution	T>TA	Heterozygous	28618T>TA	5.0%	None	Not Found
	1:70905045	Inversion	T>TA	Heterozygous	28645T>TA	5.0%	None	Not Found
	1:70904811	Substitution	T>TC	Heterozygous	28411T>TC	5.3%	None	Not Found
	1:70905047	Inversion	G>GA	Heterozygous	28647G>GA	5.3%	None	Not Found
	1:70904810	Substitution	G>GC	Heterozygous	28410G>GC	4.3%	Valine>Tyrosine	Not Found
	1:70904772	Substitution	C>CG	Heterozygous	28372C>CG	5.0%	None	Not Found
	1:70904811	Substitution	T>TC	Heterozygous	28411T>TC	5.3%	None	Not Found
	1:70905018	Substitution	T>TA	Heterozygous	28618T>TA	5.0%	None	Not Found
	1:70905045	Inversion	T>TA	Heterozygous	28645T>TA	5.0%	None	Not Found
	1:70904772	Substitution	C>CG	Heterozygous	28372C>CG	5.0%	None	Not Found

Appendix B

**Table 4:** eNOS (786) variation with different types of mutations and amino acid change in chronic myeloid leukemia (CML)

Gene	Chromosome position	Mutation	Mutation genotype	Heterozygous/homozygous	Variants	Variant percentage	Amino acid change	External database
*eNOS* (786)								
	7:150696098	Missense	A>AG	Heterozygous	8455A>AG	39.1%	Gln>Arg	Not Found
	7:150696111	Missense	T>TG	Heterozygous	8468T>TG	91.3%	Asp>Glu	dbSNP:1799983
	7:150696098	Missense	A>AG	Heterozygous	8455A>AG	39.1%	Gln>Arg	Not Found
	7:150696098	Missense	A>AG	Heterozygous	8455A>AG	39.1%	Gln>Arg	Not Found
	7:150696111	Missense	T>TG	Heterozygous	8468T>TG	91.3%	Asp>Glu	dbSNP:1799983
	7:150696052	No mutation	T>TG	Heterozygous	8409T>TG	8.7%	Trp>Gly	Not found
	7:150696098	Missense	A>AG	Heterozygous	8455A>AG	39.1%	Gln>Arg	Not Found
	7:150696111	Missense	T>TG	Heterozygous	8468T>TG	91.3%	Asp>Glu	dbSNP:1799983
	7:150696187	Splice region	G>GA	Heterozygous	8544G>GA	4.3%	None	Not Found
	7:150696098	Missense	A>AG	Heterozygous	8455A>AG	39.1%	Gln>Arg	Not Found
	7:150696111	Missense	T>TG	Heterozygous	8468T>TG	91.3%	Asp>Glu	dbSNP:1799983
	7:150696038	Substitution	G>GC	Heterozygous	8395G>GC	4.3%	Cys>Ser	Not Found
	7:150696039	Substitution	C>A	Homozygous	8396C>A	8.7%	Cys>Ser	Not Found
	7:150696052	Substitution	T>G	Homozygous	8409T>G	4.3%	Trp>Gly	Not Found
	7:150696111	Missense	T>TG	Heterozygous	8468T>TG	91.3%	Asp>Glu	dbSNP:1799983
	7:150696098	Missense	A>AG	Heterozygous	8455A>AG	39.1%	Gln>Arg	Not Found
	7:150696100	Substitution	G>GC	Heterozygous	8457G>GC	13.0%	Ala>Pro	Not Found
	7:150696111	Missense	T>G	Homozygous	8468T>G	8.7%	Trp>Gly	dbSNP:1799983
	7:150696038	Substitution	G>GC	Heterozygous	8395G>GC	4.3%	Cys>Ser	Not Found
	7:150696039	Synonymous	C>A	Homozygous	8396C>A	8.7%	Cys>Ser	Not Found
	7:150696098	Missense	A>AG	Heterozygous	8455A>AG	39.1%	Gln>Arg	Not Found
	7:150696111	Missense	T>TG	Heterozygous	8468T>TG	91.3%	Asp>Glu	dbSNP:1799983
	7:150696098	Missense	A>AG	Heterozygous	8455A>AG	39.1%	Gln>Arg	Not Found
	7:150696111	Missense	T>TG	Heterozygous	8468T>TG	91.3%	Asp>Glu	dbSNP:1799983
	7:150696014	Intron/deletion	C>T	Homozygous	8371C>T	25.0%	None	Not found
	7:150696098	Missense	A>AG	Heterozygous	8455A>AG	39.1%	Gln>Arg	Not Found
	7:150696111	Missense	T>TG	Heterozygous	8468T>TG	91.3%	Asp>Glu	dbSNP:1799983
	7:150696054	Substitution	G>GC	Heterozygous	8411G>GC	13.0%	Trp>Cys	Not Found
	7:150696055	Missense	A>AC	Heterozygous	8412A>AC	13.0%	Thr>Pro	Not Found
	7:150696098	Missense	A>AC	Heterozygous	8455A>AC	4.3%	Gln>Pro	Not Found
	7:150696111	Missense	T>G	Homozygous	8468T>G	8.7%	Trp>Gly	dbSNP:1799983
	7:150696061	Substitution	G>GA	Heterozygous	8418G>GA	4.3%	Gly>Arg	Not Found
	7:150696058	Substitution	C>CT	Heterozygous	8415C>CT	8.7%	Pro>Leu	Not Found
	7:150696059	Substitution	C>CT	Heterozygous	8416C>CT	8.7%	Pro>Leu	Not Found
	7:150696055	Substitution	A>AC	Heterozygous	8412A>AC	13.0%	Thr>Pro	Not Found
	7:150696098	Missense	A>AC	Heterozygous	8455A>AC	17.4%	Gln>Pro	Not Found
	7:150696111	Missense	T>G	Homozygous	8468T>G	8.7%	Trp>Gly	dbSNP:1799983
	7:150696111	Missense	T>TG	Heterozygous	8468T>TG	91.3%	Trp>Cys	dbSNP:1799983
	7:150696054	Substitution	G>GC	Heterozygous	8411G>GC	13.0%	Trp>Cys	Not Found
	7:150696055	Substitution	A>AC	Heterozygous	8412A>AC	13.0%	Thr>Pro	Not Found
	7:150696061	Substitution	G>GA	Heterozygous	8418G>GA	4.3%	Gly>Arg	Not Found
	7:150696098	Missense	A>AC	Heterozygous	8455A>AC	17.4%	Gln>Pro	Not Found
	7:150696099	Substitution	G>GC	Heterozygous	8456G>GC	4.3%	Gln>His	Not Found
	7:150696111	Missense	T>G	Homozygous	8468T>G	8.7%	Trp>Gly	dbSNP:1799983
	7:150696052	Substitution	T>TC	Heterozygous	8409T>TC	4.3%	Trp>Arg	Not Found
	7:150696053	Substitution	G>GC	Heterozygous	8410G>GC	4.3%	Trp>Ser	Not Found
	7:150696055	Missense	A>AC	Heterozygous	8412A>AC	13.0%	Thr>Pro	Not Found
	7:150696058	Substitution	C>CT	Heterozygous	8415C>CT	8.7%	Pro>Ser	Not Found
	7:150696060	Substitution	A>AT	Heterozygous	8417A>AT	4.3%	Pro>Pro	Not Found
	7:150696061	Substitution	G>GA	Heterozygous	8418G>GA	4.3%	Gly>Arg	Not Found
	7:150696111	Missense	T>G	Homozygous	8468T>G	8.7%	Trp>Gly	dbSNP:1799983
	7:150696022	Substitution	C>CA	Heterozygous	8379C>CA	4.3%	None	Not Found
	7:150696111	Missense	T>G	Homozygous	8468T>G	8.7%	Trp>Gly	dbSNP:1799983
	7:150696030	Substitution	A>AT	Heterozygous	8387A>AT	4.8%	None	Not Found
	7:150696032	Substitution	A>AT	Heterozygous	8389A>AT	4.8%	None	Not Found
	7:150696053	Substitution	G>GC	Heterozygous	8410G>GC	4.3%	Trp>Ser	Not Found
	7:150696055	Missense	A>AC	Heterozygous	8412A>AC	13.0%	Thr>Pro	Not Found
	7:150696060	Missense	A>AT	Heterozygous	8417A>AT	4.3%	Pro>Pro	Not Found
	7:150696061	Missense	G>GA	Heterozygous	8418G>GA	4.3%	Gly>Arg	Not Found
	7:150696064	Missense	A>AG	Heterozygous	8421A>AG	4.5%	Asn>Gly	Not Found
	7:150696065	Missense	A>AG	Heterozygous	8422A>AG	4.2%	Asn>Ser	Not Found
	7:150696093	Substitution	G>GC	Heterozygous	8450G>GC	4.3%	Leu>Leu	Not Found
	7:150696103	Missense	C>T	Homozygous	8460C>T	25.0%	Pro>Ser	Not Found
	7:150696107	Missense	A>AG	Heterozygous	8464A>AG	4.5%	Asp>Gly	Not Found
	7:150696108	Substitution	T>TG	Heterozygous	8465T>TG	91.3%	Asp>Glu	Not Found
	7:150696110	Missense	A>AC	Heterozygous	8467A>AC	4.3%	Asp>Ala	Not Found
	7:150696111	Missense	T>TG	Heterozygous	8468T>TG	91.3%	Asp>Ala	dbSNP:1799983
	7:150696118	Substitution	G>GT	Heterozygous	8475G>GT	4.3%	Glu>Glu	Not Found
	7:150696152	Substitution	A>AC	Heterozygous	8509A>AC	4.5%	Glu>Ala	Not Found
	7:150696054	Substitution	G>GC	Heterozygous	8411G>GC	13.0%	Trp>Cys	Not Found
	7:150696055	Missense	A>AC	Heterozygous	8412A>AC	13.0%	Thr>Pro	Not Found
	7:150696098	Missense	A>AC	Heterozygous	8455A>AC	17.4%	Gln>Pro	Not Found
	7:150696111	Missense	T>G	Homozygous	8468T>G	8.7%	Trp>Gly	dbSNP:1799983
	7:150696014	Intron/deletion	C>T	Homozygous	8371C>T	25.0%	None	Not Found
	7:150696054	Substitution	G>GC	Heterozygous	8411G>GC	13.0%	Trp>Cys	Not Found
	7:150696055	Missense	A>AC	Heterozygous	8412A>AC	13.0%	Thr>Pro	Not Found
	7:150696058	Substitution	C>CT	Heterozygous	8415C>CT	8.7%	Pro>Ser	Not Found
	7:150696059	Substitution	C>CT	Heterozygous	8416C>CT	8.7%	Pro>Leu	Not Found
	7:150696098	Missense	A>AC	Heterozygous	8455A>AC	17.4%	Gln>Pro	Not Found
	7:150696099	Substitution	G>GC	Heterozygous	8456G>GC	4.3%	Gln>Pro	Not Found
	7:150696111	Missense	T>G	Homozygous	8468T>G	8.7%	Trp>Gly	dbSNP:1799983
	7:150696049	Synonymous	G>GC	Heterozygous	8406G>GC	4.3%	Gly>Arg	Not Found
	7:150696050	Substitution	G>GC	Heterozygous	8407G>GC	4.3%	Gly>Ala	Not Found
	7:150696052	Substitution	T>C	Homozygous	8409T>C	4.3%	Trp>Arg	Not Found
	7:150696058	Substitution	C>CT	Heterozygous	8415C>CT	8.7%	Pro>Ser	Not Found
	7:150696059	Substitution	C>T	Homozygous	8416C>T	25.0%	Pro>Leu	Not Found
	7:150696063	Substitution	A>AG	Heterozygous	8420A>AG	4.5%	Gly>Gly	Not Found
	7:150696064	Substitution	A>AG	Heterozygous	8421A>AG	4.5%	Asn>Ala	Not Found
	7:150696065	Substitution	A>AC	Heterozygous	8422A>AC	4.5%	Asn>Thr	Not Found
	7:150696077	Substitution	A>AC	Heterozygous	8434A>AC	4.3%	Asp>Ala	Not Found
	7:150696052	Substitution	T>TC	Heterozygous	8409T>TC	4.3%	Cys>Arg	Not Found
	7:150696053	Substitution	G>GC	Heterozygous	8410G>GC	13.0%	Trp>Cys	Not Found
	7:150696054	Substitution	G>C	Heterozygous	8411G>C	8.7%	Trp>Cys	Not Found
	7:150696059	Substitution	C>T	Heterozygous	8416C>T	25.0%	Pro>Leu	Not Found
	7:150696061	Substitution	G>GA	Heterozygous	8418G>GA	4.3%	Gly>Arg	Not Found
	7:150696069	Substitution	T>TC	Heterozygous	8426T>TC	4.2%	Gly>Gly	Not Found
	7:150696096	Substitution	G>GC	Heterozygous	8453G>GC	4.3%	Leu>Leu	Not Found
	7:150696099	Substitution	G>C	Heterozygous	8456G>C	8.7%	Gln>His	Not Found
	7:150696100	Substitution	G>C	Heterozygous	8457G>C	8.7%	Ala>Pro	Not Found
	7:150696111	Missense	T>TG	Heterozygous	8468T>TG	91.3%	Asp>Ala	dbSNP:1799983
	7:150696176	Splice region	G>GC	Heterozygous	8533G>GC	10.0%	None	Not Found
	7:150696177	Substitution	A>AC	Heterozygous	8534A>AC	4.5%	None	Not Found
	7:150696178	Splice region	G>GC	Heterozygous	8535G>GC	10.0%	None	Not Found
	7:150696052	Substitution	T>TC	Heterozygous	8409T>TC	4.3%	Cys>Arg	Not Found
	7:150696053	Substitution	G>GC	Heterozygous	8410G>GC	13.0%	Trp>Cys	Not Found
	7:150696054	Substitution	G>C	Heterozygous	8411G>C	8.7%	Trp>Cys	Not Found
	7:150696061	Substitution	G>GA	Heterozygous	8418G>GA	4.3%	Gly>Arg	Not Found
	7:150696062	Substitution	G>GA	Heterozygous	8419G>GA	4.3%	Gly>Lys	Not Found
	7:150696096	Substitution	G>GC	Heterozygous	8453G>GC	4.3%	Leu>Leu	Not Found
	7:150696099	Substitution	G>C	Heterozygous	8456G>C	8.7%	Gln>His	Not Found
	7:150696100	Substitution	G>C	Heterozygous	8457G>C	8.7%	Ala>Pro	Not Found
	7:150696052	Substitution	T>TC	Heterozygous	8409T>TC	4.3%	Trp>Arg	Not Found
	7:150696053	Substitution	G>GC	Heterozygous	8410G>GC	4.3%	Trp>Ser	Not Found
	7:150696055	Missense	A>AC	Heterozygous	8412A>AC	13.0%	Thr>Pro	Not Found
	7:150696054	Substitution	G>GC	Heterozygous	8411G>GC	13.0%	Trp>Cys	Not Found
	7:150696059	Substitution	C>T	Homozygous	8416C>T	25.0%	Pro>Leu	Not Found
	7:150696061	Substitution	G>GT	Heterozygous	8418G>GT	4.3%	Gly>Gly	Not Found
	7:150696096	Substitution	G>GC	Heterozygous	8453G>GC	4.3%	Leu>Leu	Not Found
	7:150696098	Missense	A>AC	Heterozygous	8455A>AC	17.4%	Gln>Pro	Not Found
	7:150696099	Substitution	G>C	Heterozygous	8456G>C	8.7%	Gln>His	Not Found
	7:150696111	Missense	T>TG	Heterozygous	8468T>TG	91.3%	Asp>Ala	dbSNP:1799983

Appendix C

**Table 5:** eNOS (894) variation with mutations in chronic myeloid leukemia (CML)

Gene	Chromosome position	Mutation	Mutation genotype	Heterozygous/homozygous	Variants	Variant percentage	Amino acid change	External database
*eNOS* (894)								
	7:150690102	Other	G>GT	Heterozygous	2459G>GT	4.0%	None	Not found
	7:150690119	Other	C>CG	Heterozygous	2476C>CG	3.8%	None	Not found
	7:150690120	Other	G>GT	Heterozygous	2477G>GT	4.0%	None	Not found
	7:150690121	Substitution	G>GT	Heterozygous	2478G>GT	4.0%	None	Not found
	7:150690079	Substitution	C>T	Homozygous	2436C>T	4.2%	None	dbSNP:2070744
	7:150690079	Substitution	C>CT	Heterozygous	2436C>CT	4.0%	None	dbSNP:2070744
	7:150690118	Substitution	G>GT	Heterozygous	2475G>GT	4.0%	None	Not found
	7:150690079	Substitution	C>T	Homozygous	2436C>T	4.2%	None	dbSNP:2070744
	7:150690079	Substitution	C>T	Homozygous	2436C>T	4.2%	None	dbSNP:2070744
	7:150689998	Substitution	G>GT	Heterozygous	2355G>GT	4.0%	None	Not found
	7:150690079	Substitution	C>T	Homozygous	2436C>T	4.2%	None	dbSNP:2070744
	7:150689996	Substitution	C>CT	Heterozygous	2353C>CT	4.0%	None	Not found
	7:150690079	Substitution	C>T	Homozygous	2436C>T	91.3%	None	dbSNP:2070744
	7:150689998	Substitution	G>GT	Heterozygous	2355G>GT	4.0%	None	Not found
	7:150690079	Substitution	C>CT	Homozygous	2436C>CT	91.3%	None	dbSNP:2070744
	7:150690079	Substitution	C>CT	Homozygous	2436C>CT	91.3%	None	dbSNP:2070744
	7:150690079	Substitution	C>T	Homozygous	2436C>T	91.3%	None	dbSNP:2070744
	7:150690102	Substitution	G>GT	Heterozygous	2459G>GT	4.0%	None	Not found
	7:150690079	Substitution	C>T	Homozygous	2436C>T	91.3%	None	dbSNP:2070744
	7:150689998	Substitution	G>GT	Heterozygous	2355G>GT	4.0%	None	Not found
	7:150690079	Substitution	C>T	Homozygous	2436C>T	91.3%	None	dbSNP:2070744
	7:150690060	Substitution	C>CA	Heterozygous	2417C>CA	4.3%	None	Not found
	7:150690062	Substitution	T>TA	Heterozygous	2419T>TA	7.0%	None	Not found
	7:150690063	Substitution	C>CA	Heterozygous	2420C>CA	4.3%	None	Not found
	7:150690065	Substitution	A>AG	Heterozygous	2422A>AG	6.2%	None	Not found
	7:150690067	Substitution	C>CG	Heterozygous	2424C>CG	100.0%	None	Not found
	7:150690079	Substitution	C>T	Homozygous	2436C>T	91.3%	None	dbSNP:2070744
	7:150690079	Substitution	C>T	Homozygous	2436C>T	91.3%	None	dbSNP:2070744
	7:150689998	Substitution	G>GT	Heterozygous	2355G>GT	4.0%	None	Not found
	7:150689998	Substitution	G>GT	Heterozygous	2355G>GT	4.0%	None	Not found
	7:150690079	Substitution	C>CT	Homozygous	2436C>CT	91.3%	None	dbSNP:2070744
	7:150690079	Substitution	C>T	Homozygous	2436C>T	91.3%	None	dbSNP:2070744
	7:150690079	Substitution	C>CT	Homozygous	2436C>CT	91.3%	None	dbSNP:2070744
	7:150690079	Substitution	C>T	Homozygous	2436C>T	91.3%	None	dbSNP:2070744
	7:150690047	Substitution	G>GC	Heterozygous	2404G>GC	25.0%	None	Not found
	7:150690048	Substitution	C>T	Homozygous	2405C>T	4.0%	None	Not found
	7:150690062	Substitution	T>TG	Heterozygous	2419T>TG	4.2%	None	Not found
	7:150690078	Substitution	C>CA	Heterozygous	2435C>CA	4.3%	None	Not found
	7:150690079	Substitution	C>T	Homozygous	2436C>T	91.3%	None	dbSNP:2070744
	7:150689998	Substitution	G>GT	Heterozygous	2355G>GT	4.0%	None	Not found
	7:150690079	Substitution	C>CT	Heterozygous	2436C>CT	91.3%	None	dbSNP:2070744
	7:150690079	Substitution	C>T	Homozygous	2436C>T	91.3%	None	dbSNP:2070744
	7:150690079	Substitution	C>T	Homozygous	2436C>T	91.3%	None	dbSNP:2070744

Appendix D

**Table 6:** eNOS (VNTR) variation with different types of mutations in chronic myeloid leukemia (CML)

Gene	Chromosome position	Mutation	Mutation genotype	Heterozygous/homozygous	Variants	Variant percentage	Amino acid change	External database
*eNOS* (VNTR)								
	7:150694357	Substitution	G>GA	Heterozygous	6714G>GA	16.7%	None	dbSNP:3918168
	7:150694570	Substitution	C>CA	Heterozygous	6927C>CA	4.3%	None	Not Found
	7:150694571	Substitution	C>CA	Heterozygous	6928C>CA	4.3%	None	Not Found
	7:150694598	Substitution	C>CG	Heterozygous	6955C>CG	7.1%	None	Not Found
	7:150694617	Substitution	C>CA	Heterozygous	6974C>CA	4.3%	None	Not Found
	7:150694619	Substitution	C>CA	Heterozygous	6976C>CA	4.3%	None	Not Found
	7:150694620	Substitution	C>CA	Heterozygous	6977C>CA	4.3%	None	Not Found
	7:150694621	Substitution	T>TA	Heterozygous	6978T>TA	4.5%	None	Not Found
	7:150694622	Substitution	G>GA	Heterozygous	6979G>GA	16.7%	None	Not Found
	7:150694624	Substitution	G>GA	Heterozygous	6981G>GA	16.7%	None	Not Found
	7:150694357	Substitution	G>GA	Heterozygous	6714G>GA	16.7%	None	dbSNP:3918168
	7:150694368_ 7:150694394	Duplication	AGTCTA GACCTG CTGCGG GGGTGA GGA	Heterozygous	6725_6751het_ dupAGTCTAG ACCTGCTGCG GGGGTGAGGA	5.6%	None	Not Found
	7:150694395	Substitution	C>CA	Heterozygous	6752C>CA	4.3%	None	Not Found
	7:150694606	Substitution	A>G	Homozygous	6963A>G	7.1%	None	Not Found
	7:150694619	Substitution	C>CA	Heterozygous	6976C>CA	4.3%	None	Not Found
	7:150694620	Substitution	C>CA	Heterozygous	6977C>CA	4.3%	None	Not Found
	7:150694357	Substitution	G>GA	Heterozygous	6714G>GA	16.7%	None	dbSNP:3918168
	7:150694394_ 7:150694395	Insertion	AGTCTA GGACCT GCTGCG GGGGTG AGGA	Heterozygous	6751_6752het_ insAGTCTAGG ACCTGCTGCGG GGGTGAGGA	5.6%	None	Not Found
	7:150694619	Substitution	C>CA	Heterozygous	6976C>CA	4.3%	None	Not Found
	7:150694620	Substitution	C>CA	Heterozygous	6977C>CA	4.3%	None	Not Found
	7:150694621	Substitution	C>CA	Heterozygous	6978T>TA	4.5%	None	Not Found
	7:150694620	Substitution	C>CA	Heterozygous	6977C>CA	4.3%	None	Not Found
	7:150694622	Substitution	G>GA	Heterozygous	6979G>GA	16.7%	None	Not Found
	7:150694623	Substitution	T>TA	Heterozygous	6980T>TA	4.5%	None	Not Found
	7:150694620	Substitution	C>CA	Heterozygous	6977C>CA	4.3%	None	Not Found
	7:150694622	Substitution	G>GA	Heterozygous	6979G>GA	16.7%	None	Not Found
	7:150694346_ 7:150694347	Insertion	SACCTG MTGCA GGGGT GAGGA GTCTA	Heterozygous	6703_6704ins SACCTGMTG CAGGGGTGA GGAGTCTA	10.5%	None	Not Found
	7:150694337	Substitution	A>AT	Heterozygous	6694A>AT	5.3%	None	Not Found
	7:150694348	Substitution	A>AT	Heterozygous	6705A>AC	7.1%	None	Not Found
	7:150694348_ 7:150694349	Insertion	TTGMTG CAGGGG TGAGGA AGTCTAG A	Heterozygous	6705_6706ins TTGMTGCAG GGGTGAGGA AGTCTAGA	10.5%	None	Not Found
	7:150694325	Substitution	G>GC	Heterozygous	6682G>GC	5.3%	None	Not Found
	7:150694328	Substitution	G>GT	Heterozygous	6685G>GT	7.1%	None	Not Found
	7:150694385_ 7:150694386	Insertion	TGGAGC CTGCCCA GTATAGA ACTGCTG CGG	Heterozygous	6742_6743het_ insTGGAGCCT GCCCAGTATA GAACTGCTGC GG	5.6%	None	Not Found
	7:150694318	Substitution	T>TC	Heterozygous	6675T>TC	5.4%	None	Not Found
	7:150694321	Substitution	A>C	Homozygous	6678A>C	7.1%	None	Not Found
	7:150694328	Substitution	G>GT	Heterozygous	6685G>GT	4.3%	None	Not Found

Appendix E

**Table 7:** NGS with all variations and mutation types and expressed genes in chronic myeloid leukemia (CML)

Chr.	Chr. mutation	Mutation	Unknown mutation	Expressed gene	Mutation no.	Mutation position	Mutation type
X	96	94	2	CXorf36	7	INTRON	0
				GYG2	1	INTRON	0
				ASB11	1	INTRON	0
				CA5B	2	INTRON	0
				ZRSR2	3	INTRON	0
				CASK	1	INTRON	0
				RP11-342D14.	7	INTRON	0
				CYSLTR1	9	INTRON	0
				TENM1	23	INTRON	0
				ENOX2	7	INTRON	0
				LINC01201	1	INTRON	0
				MTCP1	3	INTRON	0
Chr.	Chr. mutation	Mutation	Unknown mutation	Expressed gene	Mutation no.	Mutation position	Mutation type
Y	10	10		DUX4L16	2	INTRON	0
				DUX4L17	2	INTRON	0
				DUX4L18	2	INTRON	0
				MED14P1	4	INTRON	0
Chr.	Chr. mutation	Mutation	Unknown mutation	Expressed gene	Mutation no.	Mutation position	Mutation type
1	98	69	29	LINC01786	2	INTRON	0
				CAMTA1	2	INTRON	0
				PIK3CD	1	INTRON/Splice region	0
				CASZ1	1	INTRON	0
				C1orf127	1	INTRON	0
				HMGCL	1	INTRON	0
				NCMAP	1	INTRON	0
				ZCCHC17	1	INTRON	0
				AGO3	1	INTRON	0
				PTPRF	1	INTRON	0
				PODN	1	INTRON	0
				DAB1	3	INTRON	0
				AK5	1	INTRON	0
				BCL10-AS1	1	INTRON	0
				RP11-421L21.3	2	INTRON	0
				RAP1A	1	INTRON	0
				MAGI3	2	INTRON	0
				SPAG17	1	INTRON	0
				CHD1L	1		Frameshift/deletion
				CHD1L	1	INTRON	0
				FCRL4	1	INTRON	0
				RP11-550P17.5	1	INTRON	0
				TBX19	1	INTRON	0
				PAPPA2	1	INTRON	0
				LHX4	4	INTRON	0
				LHX4	2	3UTR	0
				ACBD6	9	INTRON	0
				PLEKHA6	2	INTRON	0
				LAMB3	2	INTRON	0
				HHAT	1	INTRON	0
				HEATR1	1	INTRON	0
				CHRM3	1	INTRON	0
Chr.	Chr. mutation	Mutation	Unknown mutation	Expressed gene	Mutation no.	Mutation position	Mutation type
2	166	95	68	DNAJC27-AS1	7	INTRON	0
				HAAO	1	INTRON	0
				SNTG2	1	INTRON	0
				TSSC1	1	INTRON	0
				RNF144A	1	INTRON	0
				FAM228A	1	INTRON	0
				EFR3B	1	INTRON	0
				EMILIN1	1	0	Missense
				FAM179A	2	INTRON	0
				ALK	1	INTRON	0
				AC009499.1	2	INTRON	0
				THADA	1	INTRON	0
				CAMKMT	1	INTRON	0
				PRKCE	2	INTRON	0
				AC007682.1	1	INTRON	0
				ACYP2	1	INTRON	0
				PNPT1	1	INTRON	0
				ZNF638	4	INTRON	0
				STARD7-AS1	1	INTRON	0
				AC021188.4	1	INTRON	0
				ANKRD36	1	INTRON	0
				RANBP2	1	INTRON	0
				MERTK	1	INTRON	0
				EPB41L5	2	INTRON	0
				AC012363.4	13	INTRON	0
				AC012363.8	1	Non-coding transcription	0
				MTND4P26	14	Non-coding transcription	0
				RALB	2	INTRON	0
				AC018866.1	1	INTRON	0
				CNTNAP5	1	INTRON	0
				FAM168B	2	INTRON	0
				ITGB6	1	INTRON	0
				GCA	1	3utr	0
				ABCB11	2	INTRON	0
				MTX2	1	INTRON	0
				LINC01473	2	INTRON	0
				HECW2	1	INTRON	0
				PLCL1	1	INTRON	0
				LINC01877	2	INTRON	0
				PTH2R	1	INTRON	0
				XRCC5	1	INTRON	0
				RPL37A	1	INTRON	0
				UGT1A10	1	INTRON	0
				UBE2F	1	INTRON	0
Chr.	Chr. mutation	Mutation	Unknown mutation	Expressed gene	Mutation no.	Mutation position	Mutation type
3	121	87	34	PLCL2	2		Synonymous
				GRM7	1	INTRON	
				SGO1-AS1	1	INTRON	
				RBMS3	1	INTRON	
				LINC00693	3	INTRON	
				KRBOX1	1	INTRON	
				CDCP1	1	INTRON	
				CCR5AS	3	INTRON	
				NPRL2	1		Non-coding transcript exon
				BAP1	1	INTRON	
				CACNA2D3	1	INTRON	
				IL17RD	4	INTRON	
				FLNB	54	INTRON	
				FLNB	2		Missense
				CFAP20DC	1	INTRON	
				MAGI1	1	INTRON	
				SLC25A26	2	INTRON	
				FRMD4B	21	INTRON	
				RAB7A	6	INTRON	
				EPHB1	1	INTRON	
				CP	1	INTRON	
				SSR3	1	INTRON	
				SI	1	INTRON	
				PEX5L	1	INTRON	
				LINC01206	1	INTRON	
				MAP3K13	1	INTRON	
				TBCCD1	1	0	Missense
				TBCCD1	1	0	Missense
				P3H2	1	INTRON	
				PAK2	1	3UTR	
Chr.	Chr. mutation	Mutation	Unknown mutation	Expressed gene	Mutation no.	Mutation position	Mutation type
4	81	49	32	FGFR3	2	0	Synonymous
				FGFR3	1	0	Missense
				LRPAP1	1	INTRON	
				PPP2R2C	1	INTRON	
				SORCS2	1	INTRON	
				SORCS2	1	INTRON	
				QDPR	22	INTRON	
				RBM47	1	INTRON	
				FIP1L1	5	INTRON	
				DRID2	3	INTRON	
				RP11-729M20.1	7	INTRON	
				GSTCD	2	INTRON	
				ZGRF1	1	INTRON	
				LINC01098	1	INTRON	
				TENM3	1	INTRON	
				SORBS2	1	INTRON	
Chr.	Chr. mutation	Mutation	Unknown mutation	Expressed gene	Mutation no.	Mutation position	Mutation type
5	87	47	40	MYO10	3	INTRON	
				ADCY2	1	INTRON	
				SEMA5A	2	INTRON	
				LINC02150	1	INTRON	
				CDH9	1	3'UTR	
				CDH9	1	0	Missense
				RP11-232L2.1	1	0	Non-coding transcript exon
				LINC01340	2	INTRON	
				LINC02113	1	INTRON	
				FBXL17	1	INTRON	
				FER	1	INTRON	
				NREP	1	INTRON	
				YTHDC2	3	INTRON	
				KIF3A	1	INTRON	
				PITX1-AS1	1	INTRON	
				ANKHD1	2	INTRON	
				PPP2R2B	1	5' UTR	
				GM2A	1	0	Frameshift
				GRIA1	1	INTRON	
				GEMIN5	2	INTRON	
				CLINT1	1	INTRON	
				CTC-535M15.2	1	INTRON	
				FBXW11	4	INTRON	
				CTB-32H22.1	1	INTRON	
				CPEB4	1	INTRON	
				NSG2	2	INTRON	
				COL23A1	8	INTRON	
				ZFP2	1	INTRON	
Chr.	Chr. mutation	Mutation	Unknown mutation	Expressed gene	Mutation no.	Mutation position	Mutation type
6	43	19	24	LINC02525	1	INTRON	
				RP11-288G3.4	3	0	Non-coding transcript exon
				CASC15	1	INTRON	
				ZKSCAN3	1	INTRON	
				HLA-V	3	0	Non-coding transcript exon
				HLA-DOA	1	0	Splice region
				PKHD1	1	INTRON	
				DST	1	INTRON	
				EYS	1	INTRON	
				KCNQ5	1	INTRON	
				PLAGL1	1	INTRON	
				TULP4	1	3'UTR	
				FNDC1	1	INTRON	
				PDE10A	1	INTRON	
Chr.	Chr. mutation	Mutation	Unknown mutation	Expressed gene	Mutation no.	Mutation position	Mutation type
7	141	62	79	AQP1	1	INTRON	
					3	3'UTR	
				NME8	1	INTRON	
				VPS41	1	INTRON	
				DBNL	1	INTRON	
				SEPT7P2	2	INTRON	
				AC004870.3	5	INTRON	
				SEC61G-DT	1	INTRON	
				LANCL2	1	INTRON	
				CACNA2D1	1	INTRON	
				PPP1R9A	1	INTRON	
				MUC12	1	0	Missense
				CUX1	1	INTRON	
				PRKRIP1	10	INTRON	
				ALKBH4	4	INTRON	
				AC002463.3	1	INTRON	
				COMETT	1	INTRON	
				CFTR	1	INTRON	
				SMO	3	INTRON	
				SMO	1		Missense
				STRIP2	2	3'UTR	
				CEP41	1	3'UTR	
				CALD1	2	INTRON	
				ZNF425	7	INTRON	
				ZNF398	5	INTRON	
				RP4-555L14.4	3	INTRON	
				PTPRN2	1	INTRON	
Chr.	Chr. mutation	Mutation	Unknown mutation	Expressed gene	Mutation no.	Mutation position	Mutation type
8	101	30	71	ERICH1-AS1	1	INTRON	
				ARHGEF10	2	INTRON	
				FAM167A	1	INTRON	
				CTSB	1	5'UTR	
				EPHX2	1	INTRON	
				DCTN6	2	INTRON	
				SMARCE1P4	1		Non-coding transcript exon
				RP11-56A10.1	1	INTRON	
				CHD7	1	INTRON	
				NCOA2	10	INTRON	
				RP11-463D19.2	1	INTRON	
				OSGIN2	1	INTRON	
				CDH17	1	INTRON	
				RP11-468O2.1	1		Non-coding transcript exon
				TRAPPC9	1	INTRON	
				DENND3	1	INTRON	
				LNCOC1	1	INTRON	
Chr.	Chr. mutation	Mutation	Unknown mutation	Expressed gene	Mutation no.	Mutation position	Mutation type
9	68	47	22	KANK1	2		Missense
				SLC24A1	1	INTRON	
				CDKN2A	3	3'UTR	
				CDKN2A	3	INTRON	
				CCL27	1	0	Non-coding transcript exon
				TRPM6	1	INTRON	
				VPS13A	2	INTRON	
				PSAT1	1	INTRON	
				CENPP	1	INTRON	
				GSN	1	INTRON	
				NR6A1	2	INTRON	
				LMX1B	1	INTRON	
				NIBAN2	2	INTRON	
				NIBAN2	1	INTRON	
				SLC25A25-AS1	1	INTRON	
				SURF6	13	INTRON	
				SURF6	1	INTRON	
				SURF6	1	0	Missense
				SURF6	1	0	Synonymous
				MED22	2	3'UTR	
				MED22	1	3'UTR	
				LL09NC01-254D11.1	1	INTRON	
				VAV2	1	INTRON	
Chr.	Chr. mutation	Mutation	Unknown mutation	Expressed gene	Mutation no.	Mutation position	Mutation type
10	72	50	22	LARP48	2	INTRON	
				FRMD4A	1	INTRON	
				ST8SIA6	2	INTRON	
				NEBL	1	INTRON	
				ABL1	4	INTRON	
				C10orf68	2	INTRON	
				ZNF33B	5	INTRON	
				AGAP9	1	INTRON	
				ASAH2	1	INTRON	
				ANK3	1	INTRON	
				JMJD1C	1	INTRON	
				CTNNA3	4	INTRON	
				VPS26A	3	3'UTR	
				VPS26A	2	INTRON	
				LINC02622	1	INTRON	
				NRG3	3	INTRON	
				CCSER2	2	INTRON	
				GRID1	3	INTRON	
				SNCG	1	INTRON	
				FRA10AC1	1	INTRON	
				ENTPD1	2	INTRON	
				BTRC	1	INTRON	
				SUFU	2	INTRON	
				LINC02661	6	INTRON	
				VTI1A	1	INTRON	
				ATRNL1	4	INTRON	
				ATE1	1	INTRON	
				TACC2	6	INTRON	
Chr.	Chr. mutation	Mutation	Unknown mutation	Expressed gene	Mutation no.	Mutation position	Mutation type
11	83	57	26	HRAS	1	INTRON	
				PIDD1	1	INTRON	
				PIDD1	3	0	Missense
				MUC6	1	0	Synonymous
				MUC6	1	0	Missense
				CTSD	3	INTRON	
				KIF18A	2	INTRON	
				RCN1	7	INTRON	
				WT1	3	INTRON	
				ALX4	1	INTRON	
				OR9Q1	2	INTRON	
				AHNAK	1	INTRON	
				SLC22A6	1	INTRON	
				EHBP1L1	4	INTRON	
				KDM2A	2	INTRON	
				LINC02754	8	INTRON	
				CAPN5	2	INTRON	
				TENM4	1	INTRON	
				DLG2	3	INTRON	
				DISC1FP1	5	INTRON	
				HEPHL1	2	INTRON	
				DYNC2H1	1	INTRON	
				RP11-144G7.2	1	INTRON	
				C11orf65	1	INTRON	
				ALG6	1	INTRON	
				LINC02762	5	INTRON	
				PHLDB1	2	INTRON	
				CBL	3	INTRON	
				USP2-AS1	6	INTRON	
				NECTIN1	1	INTRON	
				GRIK4	1	INTRON	
				RP11-744N12.3	1	INTRON	
Chr.	Chr. mutation	Mutation	Unknown mutation	Expressed gene	Mutation no.	Mutation position	Mutation type
12	91	55	36	DYRK4	24	INTRON	
				ANO2	1	INTRON	
				ETV6	7	INTRON	
				PTPRO	1	INTRON	
				IRAG2	1	INTRON	
				KIF21A	1	INTRON	
				ADAMTS20	2	INTRON	
				OR8S1	3	INTRON	
				KANSL2	2	3'UTR	
				KANSL2	6	INTRON	
				KANSL2	1		Synonymous
				CCDC65	1	INTRON	
				CCDC65	1	INTRON	
				BIN2	1	INTRON	
				SLC4A8	1	INTRON	
				HOXC-AS3	1	0	Non-coding transcript exon
				RP11-968A15.8	1	INTRON	
				ITGA7	1	INTRON	
				ITGA7	1	0	Missense
				NEMP1	1	0	Missense
				NEMP1	1	INTRON	
				TAFA2	1	INTRON	
				CAPS2-AS1	2	INTRON	
				E2F7	1	INTRON	
				ANKS1B	1	INTRON	
				IGF1	14	INTRON	
				RNF10	1	INTRON	
				CABP1	1	INTRON	
				ADGRD1	1	INTRON	
Chr.	Chr. mutation	Mutation	Unknown mutation	Expressed gene	Mutation no.	Mutation position	Mutation type
13	41	20	21	TPTE2	2	INTRON	
				PAN3	1	INTRON	
				RXFP2	1	INTRON	
				NBEA	1	INTRON	
				ELF1	1	INTRON	
				GUCY1B2	1	INTRON	
				TPTE2P3	1	INTRON	
				LINC00458	1	INTRON	
				LMO7	1	INTRON	
				MYCBP2	5	INTRON	
				GPC6	1	INTRON	
				GPC6	1	INTRON	
				NALF1	2	INTRON	
				PCID2	1	INTRON	
Chr.	Chr. mutation	Mutation	Unknown mutation	Expressed gene	Mutation no.	Mutation position	Mutation type
14	25	20	5	NF1P4	3	INTRON	
				SLC7A7	4	INTRON	
				PRKD1	1	INTRON	
				CDKL1	1	INTRON	
				CNIH1	2	INTRON	
				LINC02284	4	INTRON	
				NEK9	1		Synonymous
				GALC	1	INTRON	
				RIN3	2	INTRON	
				PAPOLA	1	INTRON	
				MOK	1	INTRON	
				RCOR1	1	INTRON	
				TDRD1	1	INTRON	
Chr.	Chr. mutation	Mutation	Unknown mutation	Expressed gene	Mutation no.	Mutation position	Mutation type
15	36	32	4	CCDC32	1	INTRON	
				EBP42	1	INTRON	
				TRIM69	1	INTRON	
				WDR72	1	INTRON	
				KIF23	1	INTRON	
				MTHFS	1	INTRON	
				MTHFS	23	INTRON	
				MTHFS	1		Missense
				FANCI	1	INTRON	
				CTD-2544M6.1	1	INTRON	
Chr.	Chr. mutation	Mutation	Unknown mutation	Expressed gene	Mutation no.	Mutation position	Mutation type
16	35	19	16	RAB11FIP3	1	INTRON	
					2		
				JPT2	1	INTRON	
				NDUFB10	1	INTRON	
				TRAF7	1	INTRON	
				AJ003147.9	1	INTRON	
				LA16c-306E5.3	1	INTRON	
				SRL	2	INTRON	
					13		
					1		
				VPS35	1	INTRON	
				ADCY7	1	INTRON	
				CMTM4	3	INTRON	
				RFWD3	1	INTRON	
				ADAMTS18	1	INTRON	
				WWOX	1	INTRON	
				PLCG2	1	INTRON	
				SPG7	2	INTRON	
Chr.	Chr. mutation	Mutation	Unknown mutation	Expressed gene	Mutation no.	Mutation position	Mutation type
17	68	55	13	RAP1GAP2	1	INTRON	
				ITGAE	1		Frameshift
				ITGAE	1	INTRON	
				SMTNL2	1	3'UTR	
				CHRNE	1	3'UTR	
				INCA1	1	INTRON	
				DNAH2	12	INTRON	
				DNAH2	2	INTRON	
				ULK2	1		Synonymous
				LINC02002	1	INTRON	
				PIGS	3	INTRON	
				MYO1D	1	INTRON	
				ASIC2	1	INTRON	
				CDK12	1	INTRON	
				WIPF2	1		Synonymous
				WIPF2	1		Inframe insertion
				BRCA1	1	INTRON	
				RP11-1072C15.7	1	INTRON	
				MSI2	1	INTRON	
				TANC2	1	INTRON	
				CEP112	9	INTRON	
				ABCA9	1	INTRON	
				RAB37	2	INTRON	
				CYTH	4	INTRON	
				CEP131	2	INTRON	
				SLC38A10	1	INTRON	
				SLC25A10	1	INTRON	
				AC139099.4	1	INTRON	
Chr.	Chr. mutation	Mutation	Unknown mutation	Expressed gene	Mutation no.	Mutation position	Mutation type
18	44	33	11	RP11-172F10.1	1	INTRON	
				AKAIN1	1	INTRON	
				EBP41L3	1	INTRON	
				RP11-805F19.2	1	INTRON	
				CDH2	1	INTRON	
				KIAA1328	1	INTRON	
				MIR4527HG	6	INTRON	
				MAPK4	1	INTRON	
				RP11-671P2.1	1	INTRON	
				RP11-795H16.3	1	INTRON	
				CDH7	1	INTRON	
				LINC00908	14	INTRON	
				LINC00908	1	INTRON	
				LINC00908	1	INTRON	
				LINC00908	1	INTRON	
Chr.	Chr. mutation	Mutation	Unknown mutation	Expressed gene	Mutation no.	Mutation position	Mutation type
19	53	41	12	WDR18	1	INTRON	
				WDR18	1	0	Synonymous
				DOT1L	1	0	Missense
				TLE2	1	INTRON	
				CACTIN	1	INTRON	
				CHAF1A	1	INTRON	
				LONP1	11	INTRON	
				NFIX	2	INTRON	
				NFIX	2	INTRON	
				NCAN	1	INTRON	
				CTD-2043I16.1	2	INTRON	
				URI1	1	INTRON	
				TDRD12	2	INTRON	
				TDRD12	1	0	Missense
				TDRD12	14	0	
				ZNF540	1	INTRON	
				PCAT19	1	INTRON	
				ZNF227	1	INTRON	
				ZNF235	1	INTRON	
				ZNF285	1	INTRON	
				PVR	1	INTRON	
				CBLC	1	INTRON	
				MARK4	5	INTRON	
Chr.	Chr. mutation	Mutation	Unknown mutation	Expressed gene	Mutation no.	Mutation position	Mutation type
20	29	11	18	C20orf27	1	INTRON	
				SLC23A2	1	INTRON	
				CHMP4B	5	INTRON	
				DHX35	2	INTRON	
				SLC2A10	1	INTRON	
				MIR646HG	1		Non-coding transcript exon
Chr.	Chr. mutation	Mutation	Unknown mutation	Expressed gene	Mutation no.	Mutation position	Mutation type
21	23	21	2	IFNGR2	8	INTRON	
				CHODL	2	INTRON	
				IFNAR1	1	INTRON	
				TMEM50B	3	INTRON	
				TMEM50B	3	INTRON	
				GART	1	INTRON	
				RUNX1	1	INTRON	
				C2CD2	1	3'UTR	
				PDXK	1	3'UTR	
Chr.	Chr. mutation	Mutation	Unknown mutation	Expressed gene	Mutation no.	Mutation position	Mutation type
22	44	29	15	TPTEP1	3	INTRON	
				SMPD4P1	2	INTRON	
				BCR	1	INTRON	
				MYO188	1	INTRON	
				DEPDC5	1	INTRON	
				FOXRED2	1	3'UTR	
				ELFN2	2	INTRON	
				XPNPEP3	1	INTRON	
				TCF20	15	INTRON	
				CYB5R3	1	INTRON	
				TAFA5	1	INTRON	
